# Childhood Adversity and Incident Psychotic Experiences in Early Adulthood: Cognitive and Psychopathological Mediators

**DOI:** 10.1093/schbul/sbae023

**Published:** 2024-03-04

**Authors:** Andrea P Cortes Hidalgo, Gemma Hammerton, Jon Heron, Koen Bolhuis, Paul Madley-Dowd, Henning Tiemeier, Marinus H van IJzendoorn, Stanley Zammit, Hannah J Jones

**Affiliations:** Centre for Academic Mental Health, Bristol Medical School, University of Bristol, Bristol, UK; Department of Child and Adolescent Psychiatry/Psychology, Erasmus University Medical Centre, Rotterdam, the Netherlands; Centre for Academic Mental Health, Bristol Medical School, University of Bristol, Bristol, UK; Medical Research Council Integrative Epidemiology Unit at the University of Bristol, Population Health Sciences, Bristol Medical School, University of Bristol, Bristol, UK; Centre for Academic Mental Health, Bristol Medical School, University of Bristol, Bristol, UK; Medical Research Council Integrative Epidemiology Unit at the University of Bristol, Population Health Sciences, Bristol Medical School, University of Bristol, Bristol, UK; Department of Child and Adolescent Psychiatry/Psychology, Erasmus University Medical Centre, Rotterdam, the Netherlands; Centre for Academic Mental Health, Bristol Medical School, University of Bristol, Bristol, UK; Medical Research Council Integrative Epidemiology Unit at the University of Bristol, Population Health Sciences, Bristol Medical School, University of Bristol, Bristol, UK; Department of Child and Adolescent Psychiatry/Psychology, Erasmus University Medical Centre, Rotterdam, the Netherlands; Department of Social and Behavioural Science, Harvard TH Chan School of Public Health, Boston, USA; Research Department of Clinical, Education and Health Psychology, Faculty of Brain Sciences, UCL, London, UK; Department of Psychiatry, Monash University, Melbourne, Australia; Centre for Academic Mental Health, Bristol Medical School, University of Bristol, Bristol, UK; MRC Centre for Neuropsychiatric Genetics and Genomics, Division of Psychological Medicine and Clinical Neurosciences, School of Medicine, Cardiff University, Cardiff, UK; Centre for Academic Mental Health, Bristol Medical School, University of Bristol, Bristol, UK

**Keywords:** psychosis, trauma, ALSPAC, mediation, locus of control, childhood adversity

## Abstract

**Background and Hypothesis:**

Childhood adversity is often described as a potential cause of incident psychotic experiences, but the underlying mechanisms are not well understood. We aimed to examine the mediating role of cognitive and psychopathological factors in the relation between childhood adversity and incident psychotic experiences in early adulthood.

**Study Design:**

We analyzed data from the Avon Longitudinal Study of Parents and Children, a large population-based cohort study. Childhood adversity was measured prospectively from birth to age 11 years, mediators (anxiety, depression, external locus of control [LoC], negative symptoms) were assessed at approximately 16 years of age, and *incident* psychotic experiences were assessed at ages 18 and 24 years. Mediation was examined via the counterfactual *g*-computation formula.

**Study Results:**

In total, 7% of participants had incident suspected or definite psychotic experiences in early adulthood. Childhood adversity was related to more incident psychotic experiences (OR_adjusted_ = 1.34, 95% CI = 1.21; 1.49), and this association was partially mediated via all mediators examined (proportion mediated: 19.9%). In separate analyses for each mediator, anxiety, depression, external LoC, and negative symptoms were all found to mediate the link between adversity and incident psychotic experiences. Accounting for potential confounders did not modify our results.

**Conclusions:**

Our study shows that cognitive biases as well as mood symptomatology may be on the causal pathway between early-life adversity and the development of psychotic experiences. Future studies should determine which mediating factors are most easily modifiable and most likely to reduce the risk of developing psychotic experiences.

## Introduction

Psychotic experiences (PEs), such as hallucinations or delusional beliefs, exist in the general population as a continuum, from transient PEs to chronic and impairing psychotic disorders like schizophrenia.^1^ Although important advances have been made in the understanding of the etiology of PEs, treatment strategies for PEs are not well defined.^2^ One important potential causal factor is exposure to adverse events like abuse or neglect during childhood, which have a population-attributable fraction of around 45% for PEs in adolescence.^3^ Whilst reducing exposure to adversity in the population is a laudable aim, understanding the mechanisms by which adversity increases risk of psychosis could lead to more targeted interventions to prevent PEs onset. To date, numerous studies have examined potential mediating factors, summarized by recent systematic reviews.^4–6^ While there is consistent evidence for various psychological mediators such as dissociation and other posttraumatic stress symptoms, the evidence is inconclusive regarding cognitive biases and mood symptomatology. Among the cognitive biases, external locus of control (LoC) has received particular attention. Individuals with an external LoC are more likely to believe that events they experience are attributable to external factors, like luck or other people, rather than being outcomes over which they have control.^7^ Interestingly, the degree of externality of LoC is learnt and modifiable, and is partly culturally dependent, with a more internal LoC being more valued in Western societies.^7^ An incorrect appraisal of experiences as externally caused has been hypothesized to underlie paranoid delusional experiences.^8^ An external LoC is more common in individuals exposed to adversity during childhood,^9^ and has been associated with more PEs in adolescence and young adulthood.^7^ In fact, evidence shows that having an external LoC is a robust *longitudinal* predictor of psychotic outcomes, and is linked to worse prognosis in individuals with schizophrenia.^6^ Overall, the potential mediating effect of external LoC (and related cognitive biases) in the association between childhood adversity and PEs has been examined by few studies, offering mixed results on the evidence of mediation,^4,10^ and mostly based on cross-sectional designs. Regarding mood symptomatology, negative emotions and internalizing disorders like anxiety and depression are often observed in individuals exposed to early-life adversity,^11^ as well as in individuals with PEs.^2^ In fact, an “affective pathway to psychosis” has been postulated, such that the mood symptomatology caused by adversity is hypothesized to lead to paranoid thinking like anticipation to threat, and to biased negative interpretations of the environment and the self, which in turn result in PEs.^12^ To date, there is some evidence supporting the mediating role of internalizing problems in the association between adverse events like bullying or harsh parenting and PEs,^13,14^ and in the association between abuse and positive psychotic symptoms in patients with early psychosis.^12^ Importantly, most previous studies evaluating these mediating factors used cross-sectional designs, which can only study *prevalence* of PEs rather than *incident* cases that begin after adversity exposure, or mediator measures. Such designs limit the evaluation of a temporal order and potentially lead to biased estimates.^4^ Using prospectively-ascertained measures of adversity and mediators and studying incident PEs can thus provide more robust evidence on the causal pathway between adversity and PEs.

An additional potential mediator of the association between childhood adversity and incident PEs are negative symptoms. These include anhedonia, lack of initiative and energy, social withdrawal, alogia and a flattened affect.^15^ Originally described in the context of schizophrenia, where they are common and often disabling symptoms,^15,16^ negative symptoms are also measurable and have a distribution in the general population.^17^ Negative symptoms are associated with PEs in population-based samples,^18^ though it is unclear whether they develop as part of a common syndrome with PEs,^8^ or predate and act as risk factors for PEs and transition into psychosis.^19^ Research also supports a link between childhood adversity and subsequent negative symptoms,^20^ in which these are hypothesized to appear as a coping mechanism with cognitive and behavioral features.^21^ To date, the role of negative symptoms as *mediating factors* in the association between childhood adversity and incident PEs has not yet been explored.

Using data from a large population-based cohort study, we aimed to examine the extent to which four cognitive and psychopathological factors (anxiety, depression, external LoC, and negative symptoms), assessed during adolescence, mediate the association between childhood adversity, and incident PEs in early adulthood. Based on previous evidence, we hypothesized that childhood adversity would be associated with a higher risk of incident PEs, and that this association would be partly mediated by all mediators examined.

## Methods

### Participants

We used data from the Avon Longitudinal Study of Parents and Children (ALSPAC), a longitudinal birth cohort that recruited pregnant women resident in Avon, United Kingdom with a delivery date between April 1991 and December 1992. Further, offspring eligible to enroll were invited to participate at ages 7 and 18 years. The cohort initially included 14 541 pregnancies and, in total, 14 901 children who were alive at 1 year of age (Supplementary Methods).^22–24^ Detailed information on the data and the ALSPAC study is available on http://www.bristol.ac.uk/alspac/researchers/our-data/. All participants provided informed consent for the use of data collected in questionnaires and clinics, based on the recommendations from the ALSPAC Ethics and Law Committee at the time. The study was ethically approved by the ALSPAC Ethics and Law Committee and the Local Research Ethics Committees. Study data were collected and managed using REDCap (Research Electronic Data Capture) electronic data capture tools.^25^

We included participants who had information available on childhood adversity prior to age 11 (*N* = 11 837). Among these, 5136 participants also had information for PEs reported at age 18 years or at 24 years, and thus were included in the final study sample.

## Measures

### Childhood Adversity

As previously documented by Croft et al,^3^ our measure of childhood adversity was based on 111 questions answered by parents or by the participants themselves. Most information was collected prospectively from age 0 to 10.9 years based on multiple assessments, though participants also retrospectively reported at age 22 years their experience of emotional abuse, sexual abuse, and physical abuse during childhood. All variables were binary and were used to derive the following adversity types: physical abuse, sexual abuse, emotional abuse, emotional neglect, domestic violence, and bullying. For each adversity type, the child was considered to be exposed if there was a response of “yes” to any of the questions, and non-exposed if there was a response of “no” and the participant responded to at least 50% of the questionnaires or interviews used to measure each adversity type within each age period. In analyses, we used a measure of cumulative adversity exposure, based on the number of adversity types that the child was exposed to from age 0 to 10.9 years. This score ranged from 0 (no exposure to any of the adversity types) to 6 (exposed to all adversity types).

### Psychotic Experiences

The semi-structured Psychosis-Like Symptom Interview (PLIKSi) was used to assess PEs at age 18 and 24 years^26^ (interrater reliability of PLIKSi: κ = 0.83^27^). This interview includes 12 core questions evaluating the occurrence of hallucinations (visual and auditory), delusions (spied on, persecution, thoughts read, reference, control, grandiosity, and other), and thought inference experiences (broadcasting, insertion, and withdrawal). At age 24, tactile hallucinations were also included in the assessment.^26^ Based on the participant responses and further cross-questioning, trained interviewers rated the experiences as not present, suspected, or definitely present. Coding was based on the Schedules for Clinical Assessment in Neuropsychiatry.^28^ Interviewers recorded whether PEs had occurred since age 12 years, their frequency over the previous 6 months, and the age at which PEs first occurred. Experiences attributable to sleep or fever were coded as not present.

Our main outcome was incident PEs (Yes/No). Incident PEs were defined as PEs (including suspected or definitely present) that were not attributable to sleep or fever and with an onset from age 17 years onwards based on the age 18- and 24-year assessments. Incident PEs were considered to be present if these were reported in at least one of the two assessments. The comparator group included both those without PEs and those with PEs that had an onset before age 17. The cut-off of 17 years was selected to avoid temporal overlap between the mediators’ assessment and the occurrence of PEs.

We also examined as a secondary outcome incident PEs that were frequent (≥ monthly) or distressing (reported as quite or very distressing), given that this is a more clinically relevant phenotype. Additionally, due to inconsistency in participant responses across time, we undertook a sensitivity analysis where we recoded individuals with incident PEs as defined above as having non-incident PEs if they had been rated as having PEs on a PLIKS interview undertaken at age 12 years. We did not use this variable in the main analysis as we considered PEs measures assessed in late adolescence/early adulthood to be more reliable than measures from late childhood.

### Mediators

#### Anxiety and *Depressive Symptoms.*

At age 15.5 years, anxiety and depression were assessed with the semi-structured Development and Well-Being Assessment Interview (DAWBA).^29^ This validated instrument categorizes the likelihood of a clinical diagnosis into six bands, via computerized algorithms based on the DSM-IV and the International Classification of Diseases (ICD-10). As has been previously used,^30^ we assessed a binary measure of anxiety or depression using a threshold in the prediction bands of having a 15% or higher probability of having the respective disorder. This cut-off was selected to ensure a high probability of having the disorder while maintaining a sufficient sample size in each group. In sensitivity analyses, we additionally evaluated the robustness of our results using a different cut-off (50% or higher probability prediction band).

#### Locus of *Control.*

LoC was assessed with the self-completed Children’s Nowicki and Strickland Internal, External Scale (CNSIE)^31^ questionnaire at age 16 years. Children could answer yes/no to each of the 12 questions (eg, “When bad things happen to you is it usually someone else’s fault?”) (Supplementary table 1). A weighted sum score (range 0–12) with higher scores reflecting a more external LoC^7^ was computed when data were available for at least nine items (75% of the items). When participants had missing values for more than 25% of the answers, the score was coded as missing.

#### Negative Symptoms.

 These symptoms were self-reported using the Community Assessment of Psychic Experiences^17^ questionnaire at age 16.5 years. The questionnaire included 10 items regarding symptoms such as lack of motivation and blunted affect, assessed in a 4-point Likert-scale from “No, never” to “Yes, nearly always” (Supplementary table 2). A weighted total sum score (weighted with the number of items completed) was computed based on the 10 items available if at least eight items were completed, and higher scores reflected more negative symptoms (score range = 0–30).^30^ The Cronbach’s alpha for this scale was of .87. This instrument was previously used in the current cohort, showing associations with the schizophrenia polygenic risk score^30^ and with socially fragmented neighborhoods.^32^ Additionally, it has been shown to differentiate negative symptoms from depression (based on confirmatory factor analyses and discriminant validity).^17,33^

### Intermediate Confounder: Cannabis Use

**Fig. 1.**
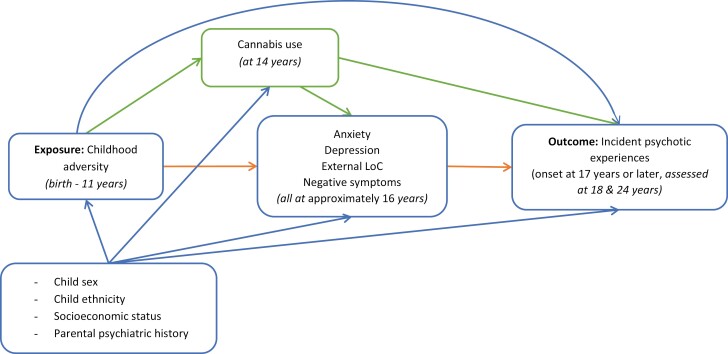
Directed acyclic graph of the association of interest.

Participants self-reported their use of cannabis and the frequency of use via a questionnaire at age 14 years. Participants who reported any frequency of use (except “only once or twice”) were categorized as cannabis-users. This dichotomous variable was included as an intermediate confounder (a variable that confounds the association between the mediators and outcome and that is additionally affected by the exposure, figure 1)^34^ given the potentially causal relation between adversity and cannabis use, and its link with subsequent PEs and the mediators examined here.^35^

### Baseline Confounders

Based on previous literature,^13,27^ we adjusted analyses for the following baseline confounders: child sex, child ethnicity, highest maternal education (reported in pregnancy), maternal and paternal occupation (reported in pregnancy), and parental psychiatric history (reported in pregnancy). Child ethnicity was based on the maternal and paternal ethnicity reported by mothers during pregnancy and was coded as “White”/“other than white.” The highest maternal education was classified into “low,” “intermediate,” and “high.”^36^ Maternal and paternal occupations were categorized as “manual” and “non-manual” occupations.^36^ Parental psychiatric history was based on maternal and paternal lifetime history of psychiatric disorders, and maternal and paternal depression during pregnancy (Supplementary Methods).

### Statistical Analyses

This project was preregistered (https://doi.org/10.17605/OSF.IO/ZW873). All analyses were performed in Stata version 16.1.^37^ Pairwise Spearman correlation coefficients were computed to examine the relation between mediators (tetrachoric correlation was used for anxiety and depression). We first examined via regression models the association between exposure and outcome, exposure and mediators, and mediators and outcome, that is, between (1) childhood adversities and incident PEs (logistic regression), (2) childhood adversity and anxiety (logistic regression), depression (logistic regression), external LoC (linear regression), and negative symptoms (linear regression), and (3) each mediator (anxiety, depression, external LoC, and negative symptoms) and incident PEs (logistic regression). These analyses were performed in unadjusted models, and also in models adjusted for baseline confounders. Analyses between the mediators and incident PEs were additionally adjusted for childhood adversity and for the intermediate confounder. There was no evidence for multicollinearity in any of these models and no evidence for violation of the assumptions of logistic and linear regression analyses.

Subsequently, we performed mediation analyses using a counterfactual framework. The goal of this analysis was to evaluate the decomposition of the causal effect of childhood adversity on incident PEs, via a direct effect and via an indirect effect^34^ mediated through anxiety, depression, external LoC, and negative symptoms (figure 1). We present an unadjusted model, a model adjusted for all baseline confounders, and a model additionally accounting for cannabis use as an intermediate confounder. These analyses were also performed for the secondary outcome of incident distressing or frequent PEs. Mediation analyses were performed using the *g*-computation formula, which was applied using the *gformula* command.^34^ Here, the mediators, outcome and intermediate confounder are simulated, via Monte Carlo simulations (25 000 simulations), under varying values of the exposure to estimate the total causal effect, pure natural direct effect, and total natural indirect effect (see also Supplementary Methods).^34^ Normal-based confidence intervals (CIs) were obtained using 100 bootstraps.

Missing values in all variables were imputed using multivariate imputation by chained equations (command *mi impute chained*) with 50 imputations, up to the sample with data for at least one adversity type and for PEs reported at age 18 and/or 24 years (*N* = 5136). The imputation of outcome and exposure was possible given the availability of closely related auxiliary variables that could enhance the imputation algorithm (see list in the Supplementary Methods). The maximum percentage of missing values was of 33.1% in incident PEs reported at age 24 years. All results from regression and mediation analyses were pooled across imputed datasets. Non-response analysis is described in the Supplementary Material.

We performed several sensitivity analyses. First, we assessed our mediation analyses separately for each mediator to explore whether a particular factor could be explaining most of the indirect effect observed in the main analyses. Second, we modified our main outcome to account for the report of PEs at age 12 years, that is, participants with incident PEs based on the report at age 18 and/or 24 years that also reported PEs at age 12 years were coded as not having the outcome of interest. Third, we repeated our main analyses in cases with complete data to evaluate comparability with the results after multiple imputation. Fourth, we repeated the analyses separately assessing the mediation via depression and via anxiety using the 50% cut-off.

## Results

Among participants included in analyses, 57% were female, and the large majority were of white ethnicity (95.8%). In total, 45.6% had experienced at least one adversity type during childhood (0–10.9 years), 10.9% had anxiety, and 9.1% had depression at age 15.5 years. Incident PEs since age 17 years were experienced by 7.1% of the participants, and of these, 3.3% had PEs that were distressing or frequent (table 1). Correlations between mediators ranged between 0.09 and 0.56 (Supplementary table 3).

**Table 1.** Sample Characteristics

Variable	*N* (%) or Mean (*SD*)	Total *N*
Sex, female (%)	2927 (57.0)	5133
Child ethnicity, white (%)	4766 (95.8)	4976
Adversity score [range: 0–6]		4947
0	2689 (54.4)	
1	1291 (26.1)	
2	601 (12.2)	
3	254 (5.1)	
4	92 (1.9)	
5	13 (0.3)	
6	7 (0.1)	
Locus of control at 16 years [range: 0–12]	3.0 (2.0)	3540
Negative symptoms at 16.5 years [range: 0–30]	6.0 (5.2)	3540
Anxiety at 15.5 years, yes (%)	434 (10.9)	3976
Depression at 15.5 years, yes (%)	360 (9.1)	3975
Incident PEs (onset since age 17 years), yes (%)	364 (7.1)	5136
Incident PEs *distressing OR frequent* (onset since age 17 years), yes (%)	168 (3.3)	5136
Cannabis use at 14 years, yes (%)	127 (3.4)	3760
Maternal education in pregnancy (%)		4989
Low	2648 (53.1)	
Intermediate	1394 (27.9)	
High	947 (19.0)	
Maternal occupation, non-manual (%)	3470 (82.7)	4197
Paternal occupation, non-manual (%)	2648 (61.1)	4336
Parental history of psychopathology, yes (%)	1051 (24.4)	4301

*Note*: Baseline characteristics in unimputed data (total *N* = 5136).

PEs, psychotic experiences.

In regression analyses, childhood adversity was related to increased odds of incident PEs (OR [odds ratio] = 1.41, 95% CI [confidence interval] = 1.28–1.56) and this association remained after adjustment for confounders (OR_adjusted_ = 1.34, 95% CI = 1.21–1.49). Childhood adversity was also associated with anxiety (OR_adjusted_ = 1.38, 95% CI = 1.26–1.51) and depression (OR_adjusted_ = 1.31, 95% CI = 1.18–1.45), a more external LoC (*B*_adjusted_ = 0.26, 95% CI = 0.19–0.33) and more negative symptoms (*B*_adjusted_ = 0.79, 95% CI = 0.62–0.97). Similarly, we observed a relation between all mediators and a greater odds of incident PEs (Anxiety: OR_adjusted_ = 2.27, 95% CI = 1.68–3.08, depression: OR_adjusted_ = 1.69, 95% CI = 1.18–2.42, external LoC: OR_adjusted_ = 1.14, 95% CI = 1.07–1.21, and negative symptoms: OR_adjusted_ = 1.05, 95% CI = 1.02–1.07) (table 2).

**Table 2.** Associations Between Childhood Adversity, Mediators of Interest, and Incident Psychotic Experiences

Predictor	Outcome	Unadjusted		Adjusted*	
		OR (95% CI)	*P* Values	OR (95% CI)	*P* Values
Cumulative childhood adversity	Incident psychotic experiences	1.41 (1.28–1.56)	<.001	1.34 (1.21–1.49)	<.001
Mediators of interest					
Anxiety		2.51 (1.89–3.32)	<.001	2.27 (1.68–3.08)	<.001
Depression		2.03 (1.46–2.83)	<.001	1.69 (1.18–2.42)	.004
External locus of control		1.18 (1.11–1.25)	<.001	1.14 (1.07–1.21)	<.001
Negative symptoms		1.06 (1.04–1.08)	<.001	1.05 (1.02–1.07)	<.001
Cumulative childhood adversity	Anxiety	1.41 (1.30–1.54)	<.001	1.38 (1.26–1.51)	<.001
	Depression	1.35 (1.23–1.49)	<.001	1.31 (1.18–1.45)	<.001

	Outcome	*B* (95% CI)	*P* Values	*B* (95% CI)	*P* Values
	External locus of control	0.30 (0.23–0.37)	<.001	0.26 (0.19–0.33)	<.001
	Negative symptoms	0.85 (0.67–1.02)	<.001	0.79 (0.62–0.97)	<.001

*Note*: Analyses between mediators of interest and incident psychotic experiences additionally adjusted for childhood adversity and cannabis use at 14 years. *N* = 5136 (estimates pooled based on 50 imputed datasets).

95% CI, 95% confidence interval.

*Analyses adjusted for: sex, ethnicity, highest maternal education, maternal occupation, paternal occupation, and parental history of psychopathology.

The direct and indirect effect of childhood adversity on incident PEs via all mediators examined simultaneously are presented in table 3. In the unadjusted model, there was evidence for a natural indirect effect (OR = 1.07, 95% CI = 1.05–1.10) which explained 21.1% of the total effect. The natural indirect effect remained after adjustment for confounders (OR_adjusted_ = 1.07, 95% CI = 1.04–1.09, proportion mediated: 22.6%), and after the inclusion of the intermediate confounder (cannabis use) (natural indirect effect: OR_adjusted_ = 1.06, 95% CI = 1.03–1.08, proportion mediated: 19.9%).

**Table 3.** Total, Direct, and Indirect Effect for Childhood Adversity and Incident Psychotic Experiences

	Unadjusted Model	Model 1	Model 2
	OR (95% CI)	OR (95% CI)	OR (95% CI)
Total causal effect	1.40 (1.28–1.53)	1.32 (1.20–1.46)	1.31 (1.19–1.45)
Natural direct effect	1.30 (1.19–1.43)	1.24 (1.12–1.37)	1.24 (1.12–1.37)
Natural indirect effect	1.07 (1.05–1.10)	1.07 (1.04–1.09)	1.06 (1.03–1.08)
Proportion mediated	21.1%	22.6%	19.9%

*Note*: Using 100 bootstrap samples, and normal-based confidence intervals (CI). Mediators examined: anxiety, depression, external LoC, and negative symptoms. *N* = 5136. Model 1 adjusted for: sex, ethnicity, highest maternal education, maternal occupation, paternal occupation, and parental history of psychopathology. Model 2: Model 1 + additionally accounting for cannabis use (intermediate confounder).

Additionally, results showed evidence for a natural indirect effect via all mediators when assessed separately, with the largest proportion mediated for anxiety (13.7%, natural indirect effect: OR_adjusted_ = 1.04, 95% CI = 1.02–1.06) and the smallest for depression (proportion mediated = 7.6%, natural indirect effect: OR_adjusted_ = 1.02, 95% CI = 1.01–1.04), though CIs for these overlapped (table 4). Analyses using distressing or frequent incident PEs as the outcome showed similarly strong evidence for mediation compared to the main analyses (proportion mediated = 21.7%, natural indirect effect: OR_adjusted_ = 1.07 [95% CI = 1.03–1.10]), while, analyses using the incident PEs outcome after recoding individuals who reported experiencing PEs at 12 years as “non-incident” since age 17 years showed 19.1% of the effect as being mediated, with a natural indirect effect of OR_adjusted_ = 1.04 (95% CI = 1.02–1.06) (Supplementary table 4). Finally, repeating our main analyses including only participants who had complete data and our analyses using the alternative anxiety and depression cut-offs yielded largely consistent results with the original ones (Supplementary Material, Supplementary table 5).

**Table 4.** Total, Direct, and Indirect Effect for Childhood Adversity and Incident Psychotic Experiences (Separately for Each Mediator)

	Mediator: Negative Symptoms	Mediator: External LoC	Mediator: Anxiety	Mediator: Depression
	OR (95% CI)	OR (95% CI)	OR (95% CI)	OR (95% CI)
Total causal effect	1.32 (1.20–1.45)	1.32 (1.20–1.45)	1.33 (1.21–1.47)	1.33 (1.21–1.47)
Natural direct effect	1.27 (1.15–1.41)	1.28 (1.16–1.42)	1.28 (1.16–1.41)	1.30 (1.18–1.44)
Natural indirect effect	1.03 (1.01–1.06)	1.03 (1.01–1.05)	1.04 (1.02–1.06)	1.02 (1.01–1.04)
Proportion mediated	12.0%	10.3%	13.7%	7.6%

*Note*: Using 100 bootstrap samples, and normal-based confidence intervals (CI). *N* = 5136. Model adjusted for: sex, ethnicity, highest maternal education, maternal occupation, paternal occupation, and parental history of psychopathology + additionally accounting for cannabis use (intermediate confounder).

## Discussion

In this large population-based study, we investigated whether anxiety, depression, a more external LoC, and negative symptoms were on the pathway between childhood adversity and incident PEs. We showed an association between childhood adversity and an increased incidence of PEs, as well as between adversity and greater levels of all mediators examined (ie, anxiety, depression, external LoC, and negative symptoms). Similarly, all mediators were related to a greater odds of incident PEs. The counterfactual mediation analyses showed evidence for a natural indirect effect, with anxiety, depression, external LoC, and negative symptoms explaining 21.1% of the association between adversity and incident PEs. The proportion mediated did not substantially change after accounting for the effects of cannabis use at age 14 years as an intermediate confounder, or when specifically addressing the more clinically relevant outcome of distressing or frequent incident PEs.

The relation between adversity and PEs has been widely examined, with studies showing that individuals exposed to early-life adversity have a higher risk of PEs in early adulthood.^3^ We contribute with research on the potential underlying mechanisms. In this study, we found evidence for a partial mediation of the association between cumulative childhood adversity and incident PEs via anxiety, depression, external LoC, and negative symptoms. Previous studies had similarly shown a mediating role of anxiety, depression and external LoC when examining the exposure to specific adverse events like harsh parenting or domestic violence,^13^ or when using a retrospective measure of various types of adversity.^38^ In particular, we extend the results of a previous study based on this same cohort, which showed mediation by affective symptomatology and cognitive biases in the association of childhood adversity and PEs at age 13 years.^13^ We add to this evidence by addressing PEs in *adulthood*, and by using a counterfactual method to address mediation. Additionally, while most research had used solely retrospective reports of adversity, possibly affected by recall bias,^39^ we implemented a thorough measure of cumulative adversity, based on multiple assessments and events, and primarily collected prospectively. Our assessment of multiple adversities offers a robust measure of the adversity effects, as it has been shown that the association with PEs is not specific for the type of adverse event.^3^ Previously, evidence was largely based on small studies with cross-sectional designs, which limited the assessment of a temporal order. In this study, we addressed this literature gap by assessing adversity during childhood, mediating factors in adolescence, and PEs in early adulthood. Importantly, our outcome was *incident* PEs that had an onset after the occurrence of adversity and the mediators’ assessment, something that has been rarely examined to date. Such a design helps reduce the risk of reverse causality, which might occur as individuals with PEs are at a higher risk of experiencing adversities,^40^ and of developing other psychopathology such as depression and anxiety.^41^ Our findings support the previously described relation between adversity and subsequent PEs and the mediating role of anxiety, depression, and an external LoC in this association.^5^

While research has shown that individuals with PEs often display negative symptoms such as flattened affect, avolition, and alogia,^16^ their specific role in relation to the occurrence of PEs is not well understood. These symptoms are unspecific, difficult to differentiate from depressive symptoms,^42^ and may also be observed in illnesses unrelated to PEs.^19^ Importantly, negative symptoms can often precede the diagnosis of psychosis for several years,^43^ and whether they develop together with PEs or act as risk factors for subsequent PEs is not yet known. In this study, we evaluated the role of negative symptoms using a mediation-based approach, and we showed that negative symptoms occurring before PEs could be on the pathway between adversity and PEs of new onset. However, based on our data, we cannot exclude the possibility that negative symptoms after adversity could be an early expression of PEs pathology. Furthermore, although the instrument used to assess negative symptoms has been shown to differentiate negative symptoms from depression (based on confirmatory factor analyses and discriminant validity),^17^ future research would need to examine whether the potential mediating role of negative symptoms is explained by affective symptoms shared with depression such as anhedonia or lack of energy, or whether it is dependent on other features like alogia and social withdrawal.

Research on mechanistic pathways underlying the association between adversity and subsequent PEs could inform the identification of targets for intervention to reduce the occurrence of PEs. For example, the mediation observed via an external LoC is of particular interest, given that the LoC orientation might be shaped by the individual’s environment and interactions with others, and research has shown that psychotherapeutic interventions could effectively modify it.^44^ Furthermore, the externality of LoC differs across ethnicities, with Asian societies having, and giving more value to, a more external LoC compared to Western societies.^45^ Interestingly, research suggests that the association between external LoC and PEs is similar across Western and Asian populations,^7^ but evidence is still limited on whether the mediating role of LoC is affected by cultural influences. Further, symptoms like anhedonia and apathy may be difficult to identify by medical health personnel if not actively assessed, so understanding their role in the onset of disorders could highlight the need for active screening for such symptoms. The current and previous studies provide growing evidence for mediation via different pathways, including PTSD and dissociation, cognitive biases, affective-related symptomatology, and possibly negative symptoms.^4^ Future research will need to replicate our findings using similarly large samples and prospective data assessments, and elucidate to what extent these pathways are independent of each other. Also of interest will be the prospective collection of information on negative beliefs related to the adverse events experienced, as these may underlie the development of (specific) psychopathological symptoms.^46^ Finally, given the ethnic composition of our study sample,^22^ results may not be directly generalizable to other populations. Thus, subsequent studies need to evaluate the relevance of specific mediation factors with a cross-cultural and inclusive approach.

Our results need to be interpreted in the light of some limitations. First, we cannot fully evaluate the temporal sequence as assessing the initial characteristics of the mediators (ie, before the exposure to adversity) is not possible. However, we provide a methodologically sound and more robust approach than previous studies. Second, it is possible that our analyses are affected by residual confounding. For example, while adjustment for parental psychiatric history did not substantially attenuate our results, this measure may not have adequately indexed shared genetic variability that might underlie both adversity exposure and PEs occurrence.^47^ Third, some of the adversity items, as well as the mediators, were self-reported by participants,^3^ which could lead to bias due to common method variance. However, it is difficult to assess the occurrence of certain adverse events if not reported by participants themselves. Furthermore, data from caregivers was also included when possible, and the assessment of the outcome, ie, PEs, was based on a semi-structured interview, which allowed a more objective evaluation of these experiences. Fourth, we could not determine the percentage mediated by each factor independently as this is not currently possible with the counterfactual mediation method used and without a clear hypothesis on the causal ordering between the mediators. Fifth, a recent meta-analysis showed a specific strong link between neglect and negative symptoms.^20^ Unfortunately, we were not able to test mediation effects specifically for neglect as physical neglect was not available within the ALSPAC childhood adversity measure and information on emotional neglect was limited. As such, this analysis would have limited statistical power in our sample. However, this would be an interesting objective for future studies. Finally, we included some retrospectively reported adverse events. However, a previous study from this cohort showed consistent results for the association of adversity and PEs when using the original adversity measure and when using a measure that excluded the data collected at 22 years.^3^

In this study, using a rich set of data from the general population and a counterfactual mediation approach, we provide evidence for partial mediation of the association between childhood adversity and the incidence of PEs via anxiety, depression, external LoC, and negative symptoms. Our results reinforce the role of socio-environmental factors like childhood adversity in the etiology of PEs and contribute to the understanding of how PEs could develop in individuals exposed to adversity. Importantly, continuing the research on the pathways underlying this association can provide insights into mechanisms and potential intervention targets for PEs and related disorders.

## Supplementary Material

Supplementary material is available at https://academic.oup.com/schizophreniabulletin/.

sbae023_suppl_Supplementary_Tables_1-5
