# Supplementary material for: Childhood Adversity and Incident Psychotic Experiences in Early Adulthood: Cognitive and Psychopathological Mediators
Source: Schizophr Bull. 2024 Mar 4;50(4):903–12. doi: 10.1093/schbul/sbae023 (PMC11283191; doi:10.1093/schbul/sbae023)
Supplement: sbae023_suppl_Supplementary_Tables_1-5 [file sbae023_suppl_supplementary_tables_1-5.docx]

**Supplementary Material for:** **Childhood Adversity and Incident Psychotic Experiences in Early Adulthood: Cognitive and Psychopathological Mediators**

**Supplementary Tables**

**Supplementary table 1. *Locus of control (LoC).*** The items assessed in the LoC measure were:

| 1.      Do you feel that wishing can make good things happen? |
| --- |
| 2.      Are people nice to you no matter what you do? |
| 3.      Do you usually do badly in your schoolwork even when you try hard? |
| 4.      When a friend is angry with you is it hard to make that friend like you again? |
| 5.      Are you surprised when your teacher praises you for your work in school? |
| 6.      When bad things happen to you is it usually someone else's fault? |
| 7.      Is doing well in your schoolwork just a matter of "luck" for you? |
| 8.      Are you often blamed for things that just aren't your fault? |
| 9.      When you get into an argument or fight is it usually the other person's fault? |
| 10.  Do you think that preparing for things is a waste of time? |
| 11.  When nice things happen to you is it usually because of "luck"? |
| 12.  Does planning ahead make good things happen? |

**Supplementary table 2. *Negative Symptoms.*** Items included in the Negative Symptoms measure:

| 1. Have you felt that you are not much of a talker when you are chatting with other people? |
| --- |
| 1. Have you felt that you experience few or no emotions at important events, such as on your birthday? |
| 1. Have you felt that you are lacking in motivation when you have to do things? |
| 1. Have you felt that you are spending all your days doing nothing? |
| 1. Have you felt that you are lacking 'get up and go'? |
| 1. Have you felt that you have only a few hobbies or interests? |
| 1. Have you felt that you have no interest to be with other people? |
| 1. Have you felt that you are not a very lively person? |
| 1. Have you felt that you are neglecting your appearance or personal hygiene? |
| 1. Have you felt that you can never get things done? |

| Supplementary table 3. Correlation between mediators | | |  |  |
| --- | --- | --- | --- | --- |
|  | External locus of control | Negative symptoms | Anxiety |  |
| Negative symptoms | 0.33 | 1 |  |  |
| Anxiety | 0.16 | 0.19 | 1 |  |
| Depression | 0.09 | 0.15 | 0.56 |  |
| Pairwise Spearman correlation between mediators (except for Anxiety and Depression, in which tetrachoric correlation was used). N range = 3034 – 3975. All correlation p-values < 0.05. | | | |  |
|  |  |  |  |  |
|  |  |  |  |  |

| Supplementary table 4. Analyses of total, direct, and indirect effect for childhood adversity and additional measures of incident psychotic experiences at age 18-24 years | | |  |
| --- | --- | --- | --- |
|  | Outcome: Distressing/frequent incident psychotic experiences | Outcome: Incident psychotic experiences recoding individuals reporting PEs at age 12 years as “non-incident” |  |
|  | **OR (95% CI)** | **OR (95% CI)** |  |
| Total causal effect | 1.35 (1.17 to 1.56) | 1.23 (1.10 to 1.37) |  |
| Natural direct effect | 1.27 (1.10 to 1.46) | 1.18 (1.06 to 1.31) |  |
| Natural indirect effect | 1.07 (1.03 to 1.10) | 1.04 (1.02 to 1.06) |  |
| Proportion mediated | 21.7% | 19.1% |  |
| Using 100 bootstrap samples, and normal-based confidence intervals (CI). N = 5136 | | |  |
| Model adjusted for: sex, ethnicity, highest maternal education, maternal occupation, paternal occupation, and parental history of psychopathology + additionally accounting for cannabis use (intermediate confounder). | | |  |
|  |  |  |  |
|  |  |  |  |
|  | | |  |

| Supplementary table 5. Total, direct, and indirect effect for childhood adversity and incident psychotic experiences (separately for anxiety and depression) | | |  |
| --- | --- | --- | --- |
|  | Mediator:  Anxiety (based on 50% cutoff) | Mediator:  Depression (based on 50% cutoff) |  |
|  | **OR (95% CI)** | **OR (95% CI)** |  |
| Total causal effect | 1.33 (1.20 to 1.46) | 1.33 (1.20 to 1.46) |  |
| Natural direct effect | 1.31 (1.18 to 1.44) | 1.32 (1.19 to 1.45) |  |
| Natural indirect effect | 1.01 (1.00 to 1.03) | 1.01 (1.00 to 1.02) |  |
| Proportion mediated | 5.0% | 2.8% |  |
| Using 100 bootstrap samples, and normal-based confidence intervals (CI). N = 5136 | | |  |
| Model adjusted for: sex, ethnicity, highest maternal education, maternal occupation, paternal occupation, and parental history of psychopathology + additionally accounting for cannabis use (intermediate confounder) | | |  |
|  |  |  |  |

| Supplementary table 6. Non-response analyses | |  |  |  |  |
| --- | --- | --- | --- | --- | --- |
|  | **Included in analyses**  **(total N = 5136)** | | **Participants with adversity data but no information for psychotic experiences at age 18 OR 24 years (total N = 6701)** | |  |
|  |  |  |  |  | ***P value*** |
| **Variable** | **N (%)** | **Total N** | **N (%)** | **Total N** |  |
| Sex, female (%) | 2927 (57.0) | 5133 | 2795 (41.8) | 6690 | < 0.001 |
| Child ethnicity, white (%) | 4766 (95.8) | 4976 | 5879 (95.3) | 6167 | 0.253 |
| Adversity score [range: 0-6] |  | 4947 |  | 5665 | < 0.001 |
| 0 | 2689 (54.4) |  | 3232 (57.1) |  |  |
| 1 | 1291 (26.1) |  | 1230 (21.7) |  |  |
| 2 | 601 (12.2) |  | 769 (13.6) |  |  |
| 3 | 254 (5.1) |  | 249 (4.4) |  |  |
| 4 | 92 (1.9) |  | 136 (2.4) |  |  |
| 5 | 13 (0.3) |  | 30 (0.5) |  |  |
| 6 | 7 (0.1) |  | 19 (0.3) |  |  |
| Maternal education in pregnancy (%) |  | 4989 |  | 6202 | < 0.001 |
| Low | 2648 (53.1) |  | 4392 (70.8) |  |  |
| Intermediate | 1394 (27.9) |  | 1227 (19.8) |  |  |
| High | 947 (19.0) |  | 583 (9.4) |  |  |
| Maternal occupation, non-manual (%) | 3470 (82.7) | 4197 | 3504 (75.5) | 4644 | < 0.001 |
| Paternal occupation, non-manual (%) | 2648 (61.1) | 4336 | 2427 (47.3) | 5133 | < 0.001 |
| Parental psychiatric history, yes (%) | 1051 (24.4) | 4301 | 1501 (30.2) | 4969 | < 0.001 |
| P-values based on chi-square tests (all variables are categorical) | | | | | |

**Supplementary Methods**

**Participants**

Pregnant women resident in Avon, UK with expected dates of delivery between 1st April 1991 and 31st December 1992 were invited to participate. The initial number of pregnancies enrolled was 14,541 (14,203 unique mothers), resulting in 13,988 children who were alive at 1 year of age. Additionally, children eligible to enrol who had not joined the study originally were invited to participate at age seven years and therefore, the total sample size, using any data collected after the age of seven years is of 15,447 pregnancies (14,833 unique women (G0 mothers)). Of these, 14,901 children were alive at 1 year of age.

In total, 12,113 G0 partners have been in contact with the study, and 3,807 G0 partners are currently enrolled.

**Measures**

***Baseline confounders***

Child ethnicity was based on the maternal and paternal ethnicity reported by mothers during pregnancy and was coded as “White”/ “other than white”. The ethnicity category “other than white” included the following ethnic groups: black/Caribbean, black/African, other black, Indian, Pakistani, Bangladeshi, Chinese, and any other ethnic group. Children were considered of “other than white” ethnic origin if either parent was of “other than white” ethnicity. The highest maternal education, reported by mothers during pregnancy, was classified into “low” (Certificate of secondary education, Vocational or Ordinary (O-) level, which are qualifications generally attained at 16 years), “intermediate” (advanced (A-) level, subject-specific degrees, often achieved at 18 years), and “high” (university degree or higher).^1^ Maternal and paternal occupations were based on reports during pregnancy, assessed with the UK Registrar General’s classification, and categorized as “manual” (unskilled, semi-skilled manual, and skilled manual occupations) and “non-manual” occupations (skilled non-manual, managerial, technical, professional occupations).^1^ Parental psychiatric history was assessed as a dichotomous measure (yes/no) based on maternal and paternal reports of lifetime history of psychiatric disorders, and a measure of maternal and paternal depression during pregnancy. Children were considered to have a parent with a psychiatric history if 1) there was a positive lifetime history of psychiatric disorders reported by mothers or fathers during pregnancy, or if 2) mothers or fathers had depression during pregnancy. Depression during pregnancy was measured with the the Edinburgh Postnatal Depression Scale (EPDS)^2^ and dichotomized based on a cut-off of 13.^3,4^

**Statistical analyses**

**Effects assessed in gformula**. Under the g-computation formula, the *total causal effect* (TCE) is the comparison between the incident psychotic experiences that occur if, 1) contrary to fact, all children were exposed to one adverse event *more* than the actually observed exposure, vs 2) if the observed exposure to adversities was left unchanged. Considering X the exposure, M the mediator(s) and Y the outcome:

TCE = log{E[Y{X+1,M(X+1)}]/(1-E[Y{X+1,M(X+1)}])}-log{E[Y{X,M(X)}]/(1-E[Y{X,M(X)}])}

The *pure natural direct effect* (PNDE) aims to measure the direct effect of adverse events on incident psychotic experiences that is not mediated via the mediators examined here. It is measured as the comparison between the incident psychotic experiences that occur if, 1) contrary to fact, all children were exposed to one adverse event *more* than the actually observed exposure, BUT the level of the mediators (anxiety, depression, external LoC, and negative symptoms) were those actually observed; vs 2) the actual presence of incident psychotic experiences when the exposure and mediators are left unchanged.

PNDE = log{E[Y{X+1,M(X)}]/(1-E[Y{X+1,M(X)}])}-log{E[Y{X,M(X)}]/(1-E[Y{X,M(X)}])}

The *total natural indirect effect* (TNIE) aims to measure the effect of adverse events on incident psychotic experiences that is mediated via the mediators of interest, and it is measured as the difference between the TCE and the PNDE.

TNIE = TCE - PNDE = log{E[Y{X+1,M(X+1)}]/(1-E[Y{X+1,M(X+1)}])}-log{E[Y{X+1,M(X)}]/(1-E[Y{X+1,M(X)}])}

The *proportion mediated* (PM) corresponds to the TNIE divided by the TCE.

**Multiple imputation model.** We used as auxiliary variables well-known predictors of the variables under imputation, including measures of the variables of interest at different timepoints. We included: the polygenic risk score for schizophrenia, psychotic experiences assessed at other ages (11, 13, 14, 16, 22, and 26 years), adverse events occurring from age 11 to 17 years, external LoC at age 8 years, anxiety at 18, 22, and 24 years, depressive symptoms at 16 years, negative symptoms at 22 and 24 years, marital status, home ownership status and crowding during pregnancy, and cannabis use at 16 and 18 years. Predictive mean matching was used for imputation of non-normally distributed variables, and the adversity sum score and the parental psychiatric history variables were generated by passive imputation.

**Supplementary Results**

Analyses including only complete cases (N = 1,934) were performed using 100 bootstrap samples and 25,000 Monte Carlo simulations. Adjusting for all confounders, results showed that 20.3% of the effect was mediated, with a natural indirect effect of OR = 1.09 (95% CI = 1.06 to 1.12) and a natural direct effect of OR = 1.40 (95% CI = 1.20 to 1.64). Analyses using the 50% cutoff for anxiety and depression (N = 5136) showed a natural indirect effect of OR = 1.01 (95% CI = 1.00 to 1.03) for anxiety and a natural indirect effect of OR = 1.01 (95% CI = 1.00 to 1.02) for depression. These analyses were limited by sample size (N exposed to anxiety = 117, N exposed to depression =111) (Supplementary table 5).

**Non-response analysis.** We compared participants who were included in analyses (N = 5,136) with those lost to follow-up (i.e., who had adversity data but no information for psychotic experiences at age 18 or 24 years, N = 6,701) (Supplementary table 6). We used chi-square tests and t-tests for categorical and continuous variables, respectively. Overall, participants included in the study were more likely to be female than those lost to follow-up (study sample = 57.0%, lost-to-follow-up sample = 41.8%, *p* < 0.001). Child ethnicity did not differ between the groups (*p* = 0.253). Compared to participants lost to follow-up, those in the study sample were more likely to experience adverse events (study sample = 45.6%, lost-to-follow-up sample = 42.9%, *p* < 0.001), had more often mothers with high education levels (study sample = 19.0%, lost-to-follow-up sample = 9.4%, p < 0.001), and were less likely to have a parental history of psychopathology (study sample = 24.4%, lost-to-follow-up sample = 30.2%, *p* < 0.001).

**Additional References**

1. Alfano R, Guida F, Galobardes B, et al. Socioeconomic position during pregnancy and DNA methylation signatures at three stages across early life: epigenome-wide association studies in the ALSPAC birth cohort. *International Journal of Epidemiology.* 2018;48(1):30-44.

2. Cox JL, Holden JM, Sagovsky R. Detection of Postnatal Depression: Development of the 10-item Edinburgh Postnatal Depression Scale. *British Journal of Psychiatry.* 1987;150(6):782-786.

3. Mountain RV, Zhu Y, Pickett OR, et al. Association of Maternal Stress and Social Support During Pregnancy With Growth Marks in Children’s Primary Tooth Enamel. *JAMA Network Open.* 2021;4(11):e2129129-e2129129.

4. Paul E, Pearson R. Depressive symptoms measured using the Edinburgh Postnatal Depression Scale in mothers and partners in the ALSPAC Study: A data note [version 2; peer review: 2 approved]. *Wellcome Open Research.* 2020;5(108).
